# Two Cases of Acute Urinary Retention Associated With Acute Sarcopenia in Older Women

**DOI:** 10.7759/cureus.29451

**Published:** 2022-09-22

**Authors:** Taku Harada, Shota Nohara, Miki Sato, Kanako Kokuno, Mori Nakai

**Affiliations:** 1 General Medicine, Nerima Hikarigaoka Hospital, Tokyo, JPN; 2 Diagnostic Generalist Medicine, Dokkyo Medical University Hospital, Mibu, JPN; 3 Internal Medicine, Nerima Hikarigaoka Hospital, Tokyo, JPN

**Keywords:** hospitalization-related complications, frailty, severe sarcopenia, acute urinary retention, acute sarcopenia

## Abstract

Acute urinary retention during hospitalization is a poor prognostic predictor. Therefore, prevention of the same is important. Herein, we describe two cases of acute urinary retention in older women admitted to the hospital for acute illness, with severe sarcopenia being the only predisposing cause. Both women developed urinary problems shortly after admission. In both, acute urinary retention was preceded by an in-hospital period of poor nutritional intake and a lack of progress in rehabilitation. There was no evidence of genitourinary disorder, neurological disease, drug-induced dysuria, bone fracture, or pain. Both patients presented with severe sarcopenia and severe frailty.Although the loss of mass in the voiding muscles is difficult to detect, the possibility of dysuria as one of the complications of acute and severe sarcopenia was indicated in our patients. Of note, however, is that many patients with severe sarcopenia do not develop dysuria. Therefore, accumulating evidence on the possible association between severe sarcopenia and dysuria is needed to inform prevention.

## Introduction

Acute onset of urinary retention in older women during hospitalization correlates with physical function, ambulatory ability, and mortality [1,2]. The main causes of urinary retention in women during hospitalization are anatomical outlet obstruction and bladder dysfunction, associated with neurological disease, infection, drugs, and pain affecting dysuria [3]. There is also evidence of urinary retention being associated with polypharmacy and frailty [3,4]. However, in 20%-30% of cases of acute urinary retention that develops during hospitalization, the underlying cause is unknown [5].

Hospitalization-related complications such as functional decline and acute onset of sarcopenia are addressed as urgent issues, with a focus on prevention through nutrition and rehabilitation; this is because these complications are associated with prolonged rehabilitation and longer hospital stay [5]. The association of dysuria with functional decline [6,7] and sarcopenia [8] has previously been reported among patients in convalescent rehabilitation hospitals. However, the association between urinary retention and sarcopenia during hospitalization for an acute care condition has not been previously described.

Herein, we present two cases of acute urinary retention that developed during hospitalization in women with no prior history of dysuria at the time of hospitalization, following a period of poor caloric intake and a functional decline. Nevertheless, both patients presented with severe sarcopenia and severe frailty. Sarcopenia diagnosis and severity were based on the criteria of the 2019 consensus statement by the Asian Working Group for Sarcopenia (AWGS), defined as follows: low muscle strength, handgrip strength <28 kg for men and <18 kg for women; and low physical performance, gait speed of <1.0 m/s for a 6-m walk, short physical performance battery (SPPB) test score ≤9, and/or ≤5 repetitions of sit-to-stand in a 12 s period. The AWGS 2019 cutoffs for height-adjusted muscle mass use were as follows: dual-energy X-ray absorptiometry, <7.0 kg/m2 in men and <5.4 kg/m2 in women; and bioimpedance, <7.0 kg/m2 in men and <5.7 kg/m2 in women. According to the AWGS 2019 criteria, severe sarcopenia is defined by the presence of low muscle mass, muscle strength, and physical performance [9]. Frailty was quantified using the 9-level classification of the clinical frailty scale (CFS) [10]. Clinically, urinary retention was defined as an inability to urinate and a residual urine volume >300 mL in the urinary catheter [11].

## Case presentation

Case 1

An 82-year-old woman with Parkinson’s disease (Hoehn & Yahr stage 3) with levodopa monotherapy was urgently admitted with a diagnosis of malignant syndrome due to poor medication adherence. Before admission, the patient was independent in activities of daily living, with a CFS classification of 5. At the time of hospitalization, she was able to urinate on her own and did not have dysuria. After admission, the patient was unable to progress in terms of food intake and rehabilitation. Dysuria developed on post-admission day five, which did not improve with urapidil administration and intermittent voiding began. After an adjustment of her medication for Parkinson's disease, food intake and physical rehabilitation improved, with an increase in her Barthel Index score from 0 to 20. Intermittent voiding continued for 35 days, with recovery to independent urination thereafter. Before discharge, the patient’s CFS classification increased to seven, with a grip strength of 6.8 kg, although her independent gait capacity remained limited, with an SPPB score of 0. A bioelectrical impedance analysis (BIA) showed that the whole-body muscle mass was 4.0 kg/m2, indicative of severe sarcopenia.

Case 2

An 85-year-old woman was admitted for an ischemic stroke in a corona radiata lesion with mild right hemiplegia. Prior to admission, she was independent in her activities of daily living, with a CFS classification of 5. At the time of hospitalization, she was able to urinate on her own and did not have dysuria. On post-admission day three, the patient developed low-activity delirium and was unable to progress in terms of food intake and rehabilitation. Treatment consisted of suvorexant and ramelteon; antipsychotics with anticholinergic effects were not used. Additionally, the patient developed lower gastrointestinal bleeding from a rectal ulcer, hypoactive delirium, and anorexia, resulting in poor food intake and rehabilitation, with deterioration in physical function. On post-admission day seven, urinary retention developed, and intermittent voiding began. As the patient did not show improvement in her rehabilitation, the decision was made to transfer the patient to a rehabilitation facility. As urinary retention persisted, a urinary catheter was placed before transfer. At the time of transfer, the patient had a Barthel Index score of 40 and was able to walk with assistance, with an SPPB scale score of 5, a CFS classification of 7, and a grip strength of 12.6 kg. A BIA showed that the whole-body muscle mass was 3.4 kg/m2, indicative of severe sarcopenia.

## Discussion

Herein, we described two patients who developed urinary retention shortly after hospital admission for an acute health condition following a period of unsuccessful rehabilitation and poor nutritional intake. Urinary retention could not be explained by disease, pain affecting dysuria, or medication; however, both patients had severe frailty and sarcopenia. The association between acute urinary retention and sarcopenia during hospitalization for acute illness has not been reported.

We hypothesized that the acute urinary retention was related to the decline in physical function and bladder contractile muscles due to acute sarcopenia, similar to the mechanism of sarcopenia and dysphagia [12]. Although the loss of muscle mass of the voiding muscles was difficult to detect with BIA in our cases, severe sarcopenia was the only plausible cause. While there is an association between the presence of sarcopenia and the development of dysuria [8], most patients with severe sarcopenia do not develop acute urinary retention. Accumulating evidence regarding which patients with severe sarcopenia are more likely to have acute urinary retention, and this association is important to understand and prevent complications associated with hospitalization.

The frequency of acute sarcopenia in hospitalization is unknown, but it is defined as a change in muscle mass and muscle function within 28 days of the occurrence of a significant stressor, such as acute illness, surgery, or burn injury [5]. Sarcopenia leads to increased care, including longer hospital stays and increased rehabilitation. Physical activity and nutritional interventions are important for the prevention of acute sarcopenia, but their role is still unclear with respect to neuromuscular electrical stimulation and pharmacological interventions. Physical activity interventions should include prevention of bed rest as much as possible and promotion of rehabilitation. Regarding nutritional interventions, elderly people generally need more protein supplementation. In addition, leucine and hydroxymethyl butyrate supplementation have recently received attention [5]. In summary, to prevent sarcopenia in the management of elderly patients in acute care wards, it is important not only to treat acute illnesses well but also to promote rehabilitation and nutritional intake.

These cases suggest the possibility of acute urinary retention as a complication of acute sarcopenia. A limitation of these two case reports is that we did not measure whole-body muscle mass or evaluate sarcopenia before the onset of acute urinary retention. Therefore, it is impossible to know if sarcopenia was already present on admission. Further investigation into the prevention of sarcopenia and the relationship between sarcopenia and dysuria is warranted.

## Conclusions

We described two patients who developed severe sarcopenia and acute urinary retention after hospitalization for acute illness due to failure of rehabilitation and nutritional intake. Both hospital-onset sarcopenia and urinary retention are undesirable outcomes in the elderly; however, no other factor other than severe sarcopenia was found to be responsible for the acute urinary retention in these cases. Accumulating evidence on the association between severe sarcopenia and acute dysuria during hospitalization will be important to confirm causality and inform preventive strategies.
